# Classification and biomarker identification using gene network modules and support vector machines

**DOI:** 10.1186/1471-2105-10-337

**Published:** 2009-10-15

**Authors:** Malik Yousef, Mohamed Ketany, Larry Manevitz, Louise C Showe, Michael K Showe

**Affiliations:** 1 The Institute of Applied Research - The Galilee Society, Shefa-Amr, Israel; 2 Al-Qasemi Academic College, Baqa Algharbiya, Israel; 3 Computer Science Department, University of Haifa, Haifa, Israel; 4 Molecular Oncogenesis/Systems Biology Program, The Wistar Institute, Philadelphia, PA 19104, USA

## Abstract

**Background:**

Classification using microarray datasets is usually based on a small number of samples for which tens of thousands of gene expression measurements have been obtained. The selection of the genes most significant to the classification problem is a challenging issue in high dimension data analysis and interpretation. A previous study with SVM-RCE (Recursive Cluster Elimination), suggested that classification based on groups of correlated genes sometimes exhibits better performance than classification using single genes. Large databases of gene interaction networks provide an important resource for the analysis of genetic phenomena and for classification studies using interacting genes.

We now demonstrate that an algorithm which integrates network information with recursive feature elimination based on SVM exhibits good performance and improves the biological interpretability of the results. We refer to the method as SVM with Recursive Network Elimination (SVM-RNE)

**Results:**

Initially, one thousand genes selected by t-test from a training set are filtered so that only genes that map to a gene network database remain. The Gene Expression Network Analysis Tool (GXNA) is applied to the remaining genes to form *n *clusters of genes that are highly connected in the network. Linear SVM is used to classify the samples using these clusters, and a weight is assigned to each cluster based on its importance to the classification. The least informative clusters are removed while retaining the remainder for the next classification step. This process is repeated until an optimal classification is obtained.

**Conclusion:**

More than 90% accuracy can be obtained in classification of selected microarray datasets by integrating the interaction network information with the gene expression information from the microarrays.

The Matlab version of SVM-RNE can be downloaded from

## Background

Sample classification based on gene expression data is usually based on small numbers of samples and very large numbers of genes. Selecting those genes that are truly biologically important remains a problem in these types of studies.

Many methods to address these types of problems have been described [1-7], and they can be divided into two main categories: those that rely on filtering methods and the model-based or so-called wrapper approaches [1,3]. Pan [7] has reported a comparison of different filtering methods, highlighting similarities and differences between three main methods. The filtering methods, although faster than the wrapper approaches, are not particularly appropriate for establishing rankings among significant genes, as each gene is examined individually and correlations among the genes are not taken into account. Although wrapper methods appear to be more accurate, filtering methods are presently more frequently applied to data analysis than wrapper methods [3].

Guyon *et. al. *[8] compared the usefulness of RFE (Recursive Feature Elimination) for SVM against the "naïve" ranking on a subset of genes. The naïve ranking is just the first iteration of RFE to obtain ranks for each gene. They found that SVM-RFE is superior to SVM without RFE and also to other multivariate linear discriminant methods, such as Linear Discriminant Analysis (LDA) and Mean-Squared-Error (MSE) with recursive feature elimination.

Li and Yang [9] also compared the performance of Support Vector Machine to other algorithms (the Roccio relevance algorithm and Ridge Regression (RR)) for classifying gene expression datasets and also examined the contribution of recursive procedures to the classification accuracy. They show that the way in which the classifier penalizes redundant features in the recursive process has a strong influence on its success. Ridge Regression was superior in the datasets they examined.

Literature data mining has been used to construct networks of interacting genes and the way they form pathways to complete various biological tasks e.g., metabolic, transcriptional, signaling or differentiation and developmental programs. Many gene network models are constructed entirely from experimental studies described in the scientific literature and make up the content of databases such as KEGG, DAVID, and INGENUITY.

However, a variety of computational methods have also been considered for reconstructing gene networks from gene expression data including, for example, linear models described in Eugene et al [10]. GeneNT [11] is a computational tool that groups functionally related genes into tight clusters despite their expression dissimilarities. Bonneau and co-workers [12,13]devised a pair of programs which first bicluster genes and conditions, and then infer regulatory relationships among the genes. At present, this pair of programs has only been applied to prokaryotes. Chen *et. al. *[14] present a novel structure-learning method for gene network discovery from gene expression data. The method is based on information theory and a greedy search algorithm in Bayesian Network (BN) learning. The results show that the proposed method can identify networks that are close to the optimal structures when the constructed networks are compared to the original networks. Srinivasan *et. al. *[15] described recent progress in network research. They briefly survey available datasets in functional genomics, review methods for data integration and network alignment, and describe recent work on using network models to guide experimental validation. Djebbari and Quackenbush [16] suggest using preliminary networks derived from the literature and/or protein-protein interaction data as seeds for a Bayesian network analysis of microarray datasets. They claim that the seeded Bayesian Networks have the ability to identify high-confidence gene-gene interactions that have been validated by comparison to other sources of gene networks and pathway data.

Recently we have developed a new approach for selecting significant genes in comparative gene expression studies. This method, which we refer to as SVM-RCE[17], combines k-means, a clustering method, to identify correlated gene clusters and Support Vector Machines to identify and weight (rank) those gene clusters. Recursive cluster elimination (RCE) is applied to remove those clusters of genes that contribute the least to the classification performance. We have now extended this approach by selecting as initial clusters, groups of genes which a network algorithm has determined to be linked. In applying this approach one thousand genes selected by t-test from a training set are first filtered so that the only genes that map to the gene networks database remain. The Gene Expression Network Analysis Tool (GXNA, [18]) is applied to these genes to form *n *clusters of genes that are highly connected in the network. Linear SVM is used to classify the samples using these network clusters, and a weight is assigned to each cluster based on its importance to the classification. The least informative clusters are removed while retaining the remainder for the next step. This process is repeated until an optimal classification is obtained.

## Results

### Algorithm

**Figure 1:**
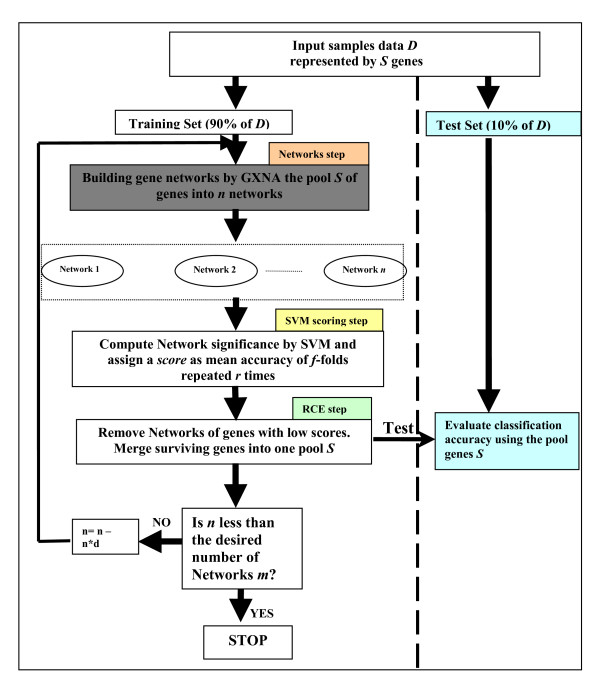
**The SVM-RNE algorithm**. A flowchart of the SVM-RNE algorithm consists of main three steps: 1) *Building Networks *for building networks of genes, 2) *SVM scoring *for assessment of significant networks and 3)*Network Elimination *to remove networks with low score.

SVM-RNE uses the Gene Expression Network Analysis Tool (GXNA)[18], a clustering method, to identify correlated gene clusters, and Support Vector Machines to identify and (rank) those gene networks (clusters) for accuracy of classification. After scoring by SVM the lowest scoring clusters are removed. The remaining clusters are merged, and the process is repeated (See Figure 1). Nacu et al [18] developed GXNA as an improvement on the method of Ideker at el [19] who propose a statistical method for scoring sub-networks and a search algorithm to determine sub-networks with high scores. GXNA is based on gene expression and prior biological information to suggest differentially expressed pathways or gene networks.

We assume a dataset D with S genes. The data is partitioned into two parts, one for training (90%) and the other (10%) for testing.

Let *X *denote a two-class training dataset consisting of ℓ samples and *S *genes. We define a score measurement for any list *S *of genes as the ability to differentiate the two classes of samples by applying linear SVM. To calculate this score we carry out a random partition of the training set *X *of samples into *f *non-overlapping subsets of equal sizes (*f*-folds). Linear SVM is trained over *f*-1 subsets and the remaining subset is used to calculate the performance. This procedure is repeated *r *times to take into account different possible partitioning. We define *Score(X(S), f, r) *as the average accuracy of the linear SVM over the data *X *represented by the S genes computed as *f*-fold cross validation repeated *r *times. We set *f *to 3 and *r *to 5 as default values. Moreover, if the *S *genes are clustered into sub-clusters of genes *S*_1_, *S*_2_, ..., *S*_*n *_then we define the *Score *(*X*(*s*_*i*_), *f, r*) for each sub-cluster while *X*(*s*_*i*_) is the data *X *represented by the genes of *S*_*i*_.

The central algorithm of the SVM-RCE method is described as a flowchart in Figure 1. It consists of three main steps applied on the training part of the data: *building gene networks *using the GXNA tool, the *SVM scoring step *for computing the *Score *(*X*(*s*_*i*_)), *f, r*) of each cluster of genes and the *RNE step *to remove clusters with low score, as follows:

### Algorithm SVM-RNE (input data *D*)

*X *= the training dataset

*S *= genes list (all the genes) or top *n_g *genes by t-test

*n *= initial number of clusters

*m *= final number of clusters

*d *= the reduction parameter

While (*n *≤ *m*) do

1. Build gene networks from *S *genes into *n *networks *S*_1_, *S*_2_, ..., *S*_*n *_using GXNA (**Building gene networks**). GXNA determines the value of *n*.

2. For each network *i *= *1*.. *n *calculate its *Score*(*X*(*s*_*i*_), *f*, *r*) (**SVM scoring step)**

3. Remove the *d% *networks with lowest score (**RNE step**)

4. Merge surviving genes again into one pool *S*

5. Test these genes on the 10% of the samples held out

6. Decrease *n *by *d%*.

The basic approach of SVM-RNE is to first group the gene expression profiles into *n *gene interaction networks, using GXNA. We have used the default parameters of GXNA. A score: *Score*(*X*(*s*_*i*_), *f*, *r*) is assigned to each of the networks by linear SVM, indicating its success at separating samples in the classification task. The *d% *networks (or *d *networks) with the lowest scores are then removed from the analysis. Steps 1 to Step 6 are repeated until the number *n *of networks is decreased to *m*.

Let *Z *denote the testing dataset. At step 4 an SVM classifier is built from the training dataset using the surviving genes S. This classifier is then tested on *Z *to estimate the performance (see Figure 1 the "Test" panel on the right side).

For the current version, the choice of *n *is determined by the GXNA tool while *m *is determined by the investigator. In this implementation, the default value of *m *is 2, indicating that the method is required to capture the top 2 significant networks (groups) of genes. However, accuracy is determined after each round of network elimination and a higher number of networks could be more accurate than the final two.

## Testing Data used for assessment of classification accuracy

We tested the SVM-RFE, SVM-RCE and SVM-RNE methods, with several datasets. The following is a brief description of these datasets.

### CTCL Datasets (I) and (II)

Cutaneous T-cell lymphoma (CTCL) refers to a heterogeneous group of non-Hodgkin lymphomas. CTCL(I) includes 18 patients and 12 controls [20] while CTCL(II) consist of 58 patients and 24 controls (Loboda et. al. unpublished). For more information about the data and preprocessing refer to [20,21].

**Lymphocyte **data is from the GXNA study [18]. This data set is related to the role of the immune system in cancer. It is derived from blood samples of 26 healthy and 30 melanoma patients. Lymphocytes were sorted according to their type into B-cells, CD4 T-cells, CD8 T-cells and NK (natural killer) cells. Gene expression data was obtained using 56 Agilent Human 1A version 2 microarrays. After removing saturated genes, there were 20901 genes left [22]

### Airway epithelial gene expression

We also re-analyzed the airway epithelial gene expression of Spira et al. [23] using SVM-RNE. The data set consists of 129 samples, 60 smokers with lung cancer and 69 smokers without lung cancer. The gene expression data was obtained using Affymetrix HG-U133A microarrays obtained from bronchial brushings. The analysis of the training set (*n *= 77) identified an 80-gene biomarker by selecting the most frequently 40 up-regulated and 40 down-regulated selected by internal cross-validation. The 80-gene biomarker identified using a weighted-voting algorithm achieved an accuracy of 83% (80% sensitivity, 84% specificity on an independent test set).

**Figure 2:**
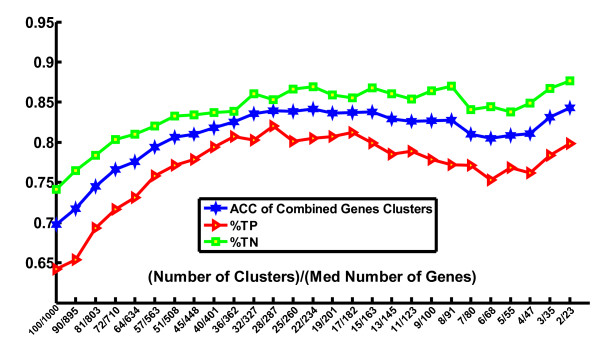
**Classification performance of SVM-RCE on the Lymphocyte data set**. All of the values are an average of 100 iterations of SVM-RCE. ACC is the accuracy, TP is the sensitivity, and TN is the specificity of the remaining genes determined on the test set. The x-axis shows the median number of clusters and number of genes in the clusters at each step.

**Figure 3:**
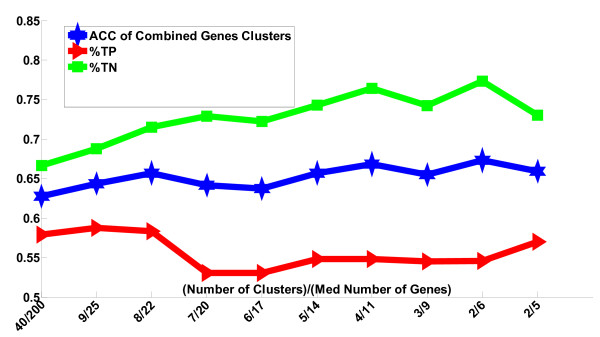
**Classification performance of SVM-RNE on the Lymphocyte data set**. All of the values are an average of 100 iterations of SVM-RNE. ACC is the accuracy, TP is the sensitivity, and TN is the specificity of the remaining genes determined on the test set. The x-axis shows the median number of genes hosted by the networks.

Table 1 summarizes the comparison of three SVM algorithms on the three lymphocyte data sets: CTCL(I), CTCL(II) and Lymphocyte. In each case, one of the clustering methods is superior in accuracy to SVM-RFE usually with a smaller gene panel. Figures 2 and 3 compare the two clustering methods applied to the lymphocyte data set. In this case, SVM-RCE exhibited superior accuracy. The range of standard deviation over different size of the networks of SVM-RNE on lymphocyte data is 0.064-0.08 while the range is 0.07-0.1 on CTCL(II) (0.09-0.14 on CTCL(I)).

**Table 1:** Summary results for the SVM-RNE, SVM-RCE and SVM-RFE algorithms.

	**CTCL(I)**			**CTCL(II)**			**Lymphocyte**		
	**c**	**g**	**ACC**	**c**	**g**	**ACC**	**c**	**g**	**ACC**

SVM-RNE	2	4	100%	2	5	91%	4	13	80%
	4	8	97%	4	8	90%	6	18	79%
	14	31	96%	14	33	90%	11	30	74%
	24	55	92%	30	69	89%			

SVM-RCE	2	8	96%	2	8	76%	2	13	96%
	3	12	96%	9	34	89%			
	9	32	97%	19	71	91%			
	15	51	97%	28	104	91%			
	32	101	96%						
							6	39	92%
							10	64	92%

SVM-RFE		9	89%		8	84%		12	81%
		32	94%		32	85%		18	79%
		102	100%		102	87%		30	77%

Classification accuracies for the three algorithms on three datasets are presented at representative steps in the course of recursive feature elimination. The number of clusters (c) and the total number of genes in the clusters (g) are shown for the steps which are presented in reverse order, i.e. the last elimination step is shown first in the table. No clusters are shown for SVM-RFE since the genes are eliminated without clustering.

SVM-RCE and SVM-RNE were applied to the airway epithelium data with 10-fold cross validation repeated 10 times (100 iterations). An accuracy of 84% with 23 genes was obtained (79% sensitivity and 87% specificity for SVM-RCE. Only 3 genes are common between the top 80 genes from SVM-RCE and the original 80 gene panel obtained by weighted voting. When SVM-RNE was used in this analysis only 408 of the 2,200 original significant genes mapped to existing networks. The performance of SVM-RNE was significantly less accurate than SVM-RCE in this case suggesting that the GXNA data base does not contain the network information related to the differential gene expression that distinguished these 2 sample classes in the airway epithelium data set.

## Implementation

SVM-RNE was written in MATLAB code (Matlab release version 7 or above). It took about 7.30 Hours to complete one experiment (10-folds repeated 10 times) on airway epithelium data (Laptop with Intel^® ^Core™ 2 Duo CPU T5670 @1.80 GHz)

## Discussion

Various methods have been used for classification studies to find the optimal subset of genes that gives the highest accuracy [24] in distinguishing members of different sample classes. With SVM-RNE, one can think of this process as a search in the gene-networks space for the *m *networks, of interacting genes, that give the highest classification accuracy. In the simplest case, the search is reduced to the identification of one or two networks that define the class differences. These might include the important up-regulated and the important down-regulated genes. While SVM-RNE and SVM-RFE are related, in that they both assess the relative contributions of the genes to the classifier, SVM-RNE assesses the contributions of groups of interacting genes instead of individual genes (SVM scoring step in Figure 1). Additionally, although both methods remove the least important genes at each step, SVM-RNE scores and removes clusters of genes, while SVM-RFE scores and removes a single or small numbers of genes at each round of the algorithm. The difference between SVM-RCE and SVM-RNE is in the way the genes are grouped: SVM-RCE uses k-means clustering, while SVM-RNE uses a network construction algorithm.

## Conclusion

In addition to providing biomarkers for distinguishing classes, an additional aim of most classification studies is to determine the biological basis for the class differences. If the expression levels of several genes on a single pathway are found to be altered, confidence in classification is increased and an understanding of the biology underlying the class differences may be enhanced. Using network fragments as units of information should make significant pathway identification easier than trying to assemble single genes into a pathway after their selection, since most genes will belong to numerous pathways.

The success of the SVM-RNE in classification studies suggests that a pathway based metric or other biological metrics to may be used to group the genes useful for classification studies and provide an alternative approach to single gene studies. The exploration of the way other factors can contribute to the classification and to the characterization of new sub-classes will be the subject of future studies [25].

## Competing interests

The authors declare that they have no competing interests.

## Authors' contributions

MY, LS and MS equally contributed to the development of the algorithm SVM-RNE while MK wrote the Matlab code for the SVM-RNE method and measured the statistical significance of the method. LM has contributed to the development of the computational method. All authors approved the manuscript.
